# Haploinsufficiency of the *folliculin* gene leads to impaired functions of lung fibroblasts in patients with Birt–Hogg–Dubé syndrome

**DOI:** 10.14814/phy2.13025

**Published:** 2016-11-15

**Authors:** Yoshito Hoshika, Fumiyuki Takahashi, Shinsaku Togo, Muneaki Hashimoto, Takeshi Nara, Toshiyuki Kobayashi, Fariz Nurwidya, Hideyuki Kataoka, Masatoshi Kurihara, Etsuko Kobayashi, Hiroki Ebana, Mika Kikkawa, Katsutoshi Ando, Koichi Nishino, Okio Hino, Kazuhisa Takahashi, Kuniaki Seyama

**Affiliations:** ^1^ Divisions of Respiratory Medicine Juntendo University Faculty of Medicine and Graduate School of Medicine Tokyo Japan; ^2^ The Study Group of Pneumothorax and Cystic Lung Diseases Tokyo Japan; ^3^ Department of Molecular and Cellular Parasitology Juntendo University School of Medicine Tokyo Japan; ^4^ Department of Pathology and Oncology Juntendo University School of Medicine Tokyo Japan; ^5^ Pneumothorax Research Center and Division of Thoracic Surgery Nissan Tamagawa Hospital Tokyo Japan; ^6^ Biomedical Research Center Laboratory of Proteomics and Biomolecular Science Juntendo University Faculty of Medicine and Graduate School of Medicine Tokyo Japan

**Keywords:** Chemotaxis, fibroblast, folliculin, haploinsufficiency, TGF‐*β*1

## Abstract

Birt–Hogg–Dubé syndrome (BHDS) is an autosomal dominant inherited disorder caused by germline mutations in the *FLCN* gene, and characterized by skin fibrofolliculomas, multiple lung cysts, spontaneous pneumothorax, and renal neoplasms. Pulmonary manifestations frequently develop earlier than other organ involvements, prompting a diagnosis of BHDS. However, the mechanism of lung cyst formation and pathogenesis of pneumothorax have not yet been clarified. Fibroblasts were isolated from lung tissues obtained from patients with BHDS (*n* = 12) and lung cancer (*n* = 10) as controls. The functional abilities of these lung fibroblasts were evaluated by the tests for chemotaxis to fibronectin and three‐dimensional (3‐D) gel contraction. Fibroblasts from BHDS patients showed diminished chemotaxis as compared with fibroblasts from controls. Expression of *fibronectin* and *TGF‐β1* was significantly reduced in BHDS fibroblasts when assessed by qPCR. Addition of TGF‐*β*1 in culture medium of BHDS lung fibroblasts significantly restored these cells' abilities of chemotaxis and gel contraction. Human fetal lung fibroblasts (HFL‐1) exhibited reduced chemotaxis and 3‐D gel contraction when *FLCN* expression was knocked down. To the contrary, a significant increase in chemotactic activity toward to fibronectin was demonstrated when wild‐type *FLCN* was overexpressed, whereas transduction of mutant *FLCN* showed no effect on chemotaxis. Our results suggest that *FLCN* is associated with chemotaxis in lung fibroblasts. Together with reduced TGF‐*β*1 expression by BHDS lung fibroblasts, a state of *FLCN* haploinsufficiency may cause lung fibroblast dysfunction, thereby impairing tissue repair. These may reveal one mechanism of lung cyst formation and pneumothorax in BHDS patients.

## Introduction

Birt–Hogg–Dubé syndrome (BHDS) is an autosomal dominant inherited disorder caused by germline mutations in the *FLCN* (folliculin) gene. The distinguishing features of BHDS are skin fibrofolliculomas, multiple lung cysts, spontaneous pneumothorax, and renal neoplasms (Birt et al. 1977). The *FLCN* gene is located on chromosome 17p11.2 and encodes a 64 kDa protein called folliculin with no characteristic functional domains (Nickerson et al. 2002). Folliculin forms a complex with novel folliculin‐interacting proteins 1 and 2 (FNIP1 and FNIP2), and 5′‐AMP‐activated protein kinase (AMPK), an important energy sensor in cells that negatively regulates mechanistic target of rapamycin (mTOR) (Baba et al. 2006; Takagi et al. 2008). The *FLCN* gene is thought to be a tumor suppressor because a second somatic mutation inactivating wild‐type *FLCN* is demonstrated in BHDS‐associated renal tumors (Vocke et al. 2005). However, fibrofolliculomas from BHDS patients do not necessarily have *FLCN* loss of heterozygosity (LOH), meaning that tumor‐like lesions of the hair follicle are evoked by a haploinsufficiency of *FLCN* (van Steensel et al. 2007). Similarly, lung cysts are likely to develop because of haploinsufficiency status of the *FLCN* gene since no neoplastic cells have been identified in cyst walls or throughout lungs. Accordingly, we theorized that investigating the effect exerted by haploinsufficiency of the *FLCN* gene on the cellular function of lungs would disclose pathogenesis of lung cyst and pneumothorax that underlie BHDS.

Lung fibroblasts are considered crucial for maintaining the integrity of alveolar structures by producing extracellular matrix proteins required for attachment, structure, and function of alveolar epithelial cells (Dunsmore and Rannels 1996). Warren et al. demonstrated that *FLCN* mRNA was strongly expressed in stromal cells within the connective tissue (Warren et al. 2004). After histopathological and morphometric analysis of the BHDS cysts, we recently reported that pulmonary cysts are likely to develop in the periacinar region, an anatomically weak site in a primary lobule, where alveoli attach to connective tissue septa (Kumasaka et al. 2014). Additionally, characteristic distribution of the BHDS cysts in the basilar medial and lateral regions on CT of the chest (Tobino et al. 2011) may imply that shear stress imposed by respiratory movement of the lungs and heartbeats participates in cyst formation. Accordingly, we hypothesized that impaired tissue repair responses or fragility of the lungs due to *FLCN* haploinsufficiency are involved in BHD cyst formation. To assess these possibilities, we performed two functional‐phenotype assays: fibroblast chemotaxis and fibroblast contraction of three‐dimensional (3‐D) collagen gels. Our aims were to evaluate the functions of primary cultured lung fibroblasts in BHDS and then to clarify how a state of haploinsufficiency of the *FLCN* gene affects lung fibroblasts.

## Materials and Methods

### Study population

Twelve patients with BHDS and 10 patients with lung cancer as a control for BHDS were included in this study (Table 1). All BHDS subjects had undergone surgery for pneumothorax, and all subjects with lung cancer were operated for tumor resection. Portion of resected lung tissues were used for a primary culture of lung fibroblasts as described below. Each subject provided written, informed consent for the acquisition of material for research. This study was approved by institutional ethics committee at Juntendo University and Tamawaga hospital.

**Table 1:** Patient characteristics of study population with BHDS

Patient no.	Age	Gender	Exon	Nucleotide change	No. and location of PTX	Lung cysts	Skin lesions	Renal tumors
1	38	M	4	c.119delG	R(5)	+	−	−
2	39	F	7	c.769_771delTCC	R(5)L(1)	+	−	−
3	48	F	9	c.932_933delCT	R(2)L(1)	+	+	−
4	38	M	11	c.1285dupC	R(10)L(5)	+	−	−
5	43	F	11	c.1285dupC	R(1)L(1)	+	−	−
6	50	M	11	c.1285dupC	R(2)L(1)	+	−	−
7	39	M	11	c.1285dupC	R(2)	+	−	−
8	62	F	11	c.1285dupC	R(3)	+	−	−
9	61	M	11	c.1285dupC	L(6)	+	−	−
10	57	F	12	c.1347_1353dupCCACCCT	R(3)L(2)	+	−	−
11	34	M	13	c.1533_1536delGATA	R(3)L(1)	+	−	−
12	38	M	13	c.1533_1536delGATA	R(3)	+	−	−

No, number; PTX, pneumothorax.

### Isolation and primary culture of lung fibroblasts

Human fetal lung fibroblasts (HFL‐1) were purchased from the American Type Culture Collection (CCL‐153, Manassas, VA). The human lung parenchymal fibroblasts were cultured from the resected lung tissues of patients with BHDS (BHDS lung fibroblasts) and patients with lung cancer (control lung fibroblasts), using the method by Holz et al. (2004). In patients with lung cancer, only lungs without visible or palpable lung metastases were used to avoid isolating cancer‐associated fibroblasts. Briefly, the portion of lung parenchymal tissue that was free of the pleural surface was minced and placed in culture with Dulbecco's modified eagle's medium (DMEM) supplemented with 10% FCS, 100 μg/mL penicillin, and 250 μg/mL streptomycin (complete medium) in a humidified atmosphere of 5% CO_2_ and passaged every 4–5 days at 1:4 ratios. They were used for chemotaxis and three‐dimensional (3‐D) collagen gel contraction assays at the fourth to fifth passages after isolation to exclude the effect of differences in passage number.

### Mutation analysis of the *FLCN* gene and LOH analysis of the *FLCN* gene‐associated region

Genomic DNA isolated from peripheral blood leukocytes was utilized to identify a germline *FLCN* mutation according to the method described previously (Gunji et al. 2007). Briefly, each exon of the *FLCN* gene was separately amplified and then screened by denaturing high‐performance liquid chromatography. When a mobility shift was detected, sequencing of the exon of concern was performed using an automated sequencer.

For LOH analysis of the *FLCN* gene‐associated region (chromosome 17p12.2), two microsatellite markers, *D17S740* and *D17S2196*, were examined according to the method described by Khoo et al. (2003).

### Chemotaxis assay

Fibroblast migration was assessed using the Boyden blindwell chamber (Neuro Probe, Inc., Gaithersburg, MD) as previously described by Nagahama et al. (2013). Briefly, serum‐free DMEM containing human fibronectin (20 μg/mL) was placed in the bottom wells of the chamber as chemoattractant. The top and bottom wells were separated by an 8 μmol/L pore polycarbonate membrane (Neuro Probe, Inc.), and medium containing fibroblasts (1 × 10^6^ cells/mL in serum‐free DMEM was loaded into the upper wells of the chamber. The chamber was incubated at 37°C in a humidified atmosphere of 5% CO_2_ for 8 h to allow the cells to migrate. The adherent cells on the upper surface of the membrane were scraped away. The membrane was then fixed, stained with DiffQuick (Sysmex, Kobe, Japan), and mounted on a glass slide for microscopic examination. Migration was assessed by counting the number of cells in five high‐power fields. Each assay was performed in triplicate.

### Three‐dimensional collagen gel contraction assays

The fibroblast‐mediated 3‐D collagen gel contraction was measured in serum‐free DMEM using a modification of the method developed by Bell et al. (1979)) as described Kamio et al. (2007). Subconfluent fibroblasts were detached with trypsin‐EDTA (0.05% trypsin, 0.53 mmol/L EDTA‐4Na; GIBCO, Grand Island, NY) and were resuspended in serum‐free DMEM. Collagen gels were prepared by mixing the appropriate amount of Type I collagen (rat tail tendon collagen), distilled water, 4 × concentrated DMEM, and cell suspension, so that the final mixture resulted in 0.75 mg/mL of collagen, 3 × 10^5^ fibroblasts/mL gel, and a physiologic ionic strength of 1 × DMEM, and a pH of 7.4. A 500 μL portion of the gel solution was then casted into each well of a 24‐well tissue culture plate with a 2 cm^2^ growth area. After gelation, the gels were released from the surface of the culture well using a sterile spatula. They were then transferred into 60 mm tissue culture dishes (three gels in each dish) containing 5 mL of serum‐free DMEM with or without designated reagents and incubated at 37°C, 5% CO_2_ for 3 days. The ability of the fibroblasts to contract the floating gels was determined by quantifying the area of the gels daily using an LAS4000 image analyzer (GE Healthcare Bio‐Science AB, Uppsela, Sweden). Data are expressed as the percentage of gel area compared with the original gel size. Fibronectin and TGF‐*β*1 production in the culture media were determined by ELISA. Human fibronectin immunoassay (Assaypro, Charles, MO) and human TGF‐*β*1 immunoassay (R&D Systems, Minneapolis, MN) were utilized according to the manufacturer's instructions.

### Quantitative real‐time reverse transcription PCR (qRT‐PCR)

Total RNAs isolated from the primary human lung parenchymal fibroblasts and HFL‐1 cell lines were purified using mirVana^™^ miRNA Isolation Kit (Applied Biosystems, Carlsbad, CA) according to the manufacturer's protocols. Total RNAs (2 μg) were reverse‐transcribed by ThermoScript^™^ RT‐PCR System (Invitrogen, Carlsbad, CA). qRT‐PCR was carried out with Fast SYBR Green Master Mix (Applied Biosystems, Carlsbad, CA) according to the manufacturer's protocols. The following program was run: holding at 95°C for 20 sec, amplification by 40 cycles (denaturation 95°C for 3 sec, annealing and extension at 60°C for 30 sec), and melt‐curve analysis. All reactions were run in triplicate using the *β‐actin* and *GAPDH* genes as internal controls. The primers that were specific for the genes were as follows: *FLCN* forward, 5′‐GAGCCTGAGCTGTGAGGTCT‐3′; *FLCN* reverse, 5′‐GAAGGTGTGGCTGAACACAA‐3′; TGF‐*β*1 *(TGFB1)* forward, 5′‐CAACAATTCCTGGCGATACCT‐3′; TGF‐*β*1 *(TGFB1)* reverse, 5′‐GCTAAGGCGAAAGCCCTCAAT‐3′; Fibronectin *(FN)* forward, 5′‐ GAAGCCGAGGTTTTAACTGC ‐3′; Fibronectin *(FN)* reverse, 5′‐ ACCCACTCGGTAAGTGTTCC‐3′; Collagen 1 *(COL1A1)* forward, 5′‐ GTCGAGGGCCAAGACGAAG ‐3′; and Collagen 1 *(COL1A1)* reverse, 5′‐ CAGATCACGTCATCGCACAAC ‐ 3′.

### Antibodies

Production and use of polyclonal rabbit anti‐FLCN C1 antibody was previously reported by Okimoto et al. (2004). Anti‐*β*‐actin antibody and anti‐FLAG antibody (M1) were purchased from Sigma‐Aldrich (St. Louis, MO).

### Western blotting

The cells were lysed with buffer (2% SDS, 50 mmol/L Tris–HCl, pH 6.8, 10% glycerol) containing protease inhibitors (Thermo Fisher Scientific, Waltham, MA) and phosphatase inhibitors (Thermo Fisher Scientific). Western blotting analysis was carried out as described previously (Takahashi et al. 2001). Briefly, equivalent amounts of protein were separated by SDS‐PAGE and transferred to a PVDF membrane (Millipore, Bedford, MA). The membrane was probed with rabbit polyclonal antibody (polyAb) against folliculin (Okimoto et al. 2004) or rabbit monoclonal antibody (mAb) against a FLAG peptide as the first antibody, followed by the peroxidase‐conjugated secondary antibody. The binding of a primary antibody was visualized, and the images were analyzed with the enhanced chemiluminescence system (Amersham Pharmacia Biotech, Buckinghamshire, UK). The band intensities of western blots were measured using the software Quantity One version 4.6.7 (Bio‐Rad, Hercules, CA).

### Plasmid construction and transfection

For establishing the permanent expression of a transduced gene in mammalian cells, the pLenti6/V5‐D‐TOPO^®^ expression vector was used. Wild‐type *FLCN* expression vector was generated from N‐terminal FLAG‐tagged FLCN pCAG‐GS expression vector (Takagi et al. 2008) using pLenti6/V5 Directional TOPO^®^ Cloning Kit (Invitrogen) according to the manufacturer's protocols. A mutated *FLCN* (c.1285dupC) expression vector was generated from wild‐type *FLCN* expression vector using KOD‐Plus Mutagenesis Kit (TOYOBO, Osaka, Japan). Wild‐type *FLCN* or mutant *FLCN* (c.1285dupC) was transduced into HFL‐1 cell line using ViraPower Lentiviral expression system (Invitrogen) following the manufacturer's protocols. A lentiviral shRNA vector targeting *FLCN* was generated by inserting stranded oligonucleotides (shFLCN1, forward sequence 5′‐ CCGGCTCTCAGCAAGTACGAGTTTGCTCGAGCAAACTCGTACTTGCTGAGAGTTTTTG‐3′ and shFLCN2, forward sequence 5′‐ CCGGGATGGAGAAGCTCGCTGATTTCTCGAGAAATCAGCGAGCTTCTCCATCTTTTTG‐3′) into TRC2‐pLKO‐puro Vector (Sigma‐Aldrich). HFL‐1 cells were infected with the *FLCN* shRNA vectors and selected with puromycin (5 μg/mL).

### Immunofluorescence staining and confocal microcopy

The human lung fibroblast cells were plated at 0.45–1.5 × 10^5^ cells in a 35 mm glass bottom dish with Advanced TC surface (Greiner Bio‐One GmbH, Frickenhausen, Germany) that is precoated with ReproCoat^®^ (ReproCell Inc., Yokohama, Japan). After incubation at 37°C overnight, serum‐starved condition was achieved by replacing the culture medium with serum‐free medium 24 h, and followed by 2‐h stimulation with 10% FCS including medium. Cells were fixed and permeabilized with 4% paraformaldehyde (Wako Pure Chemical Industries, Osaka, Japan)/0.25% Triton X‐100 (Sigma‐Aldrich) in PBS for 30 min at 4°C, and then washed three times with 0.1% bovine serum albumin (BSA) (Sigma‐Aldrich) in PBS for 5 min each. Cells were immunostained with rhodamine‐labeled phalloidin (Molecular Probes, Eugene, OR), or mouse anti‐paxillin antibody (BD biosciences, Franklin Lakes, NJ) for 1 h at room temperature. Then, the cells were washed with 0.1% BSA in PBS three times for 5 min each. For paxillin staining, cells were further incubated with Alexa Fluor 488‐conjugated goat anti‐mouse secondary antibodies (Molecular Probes) for 1 h at room temperature. Cells were mounted with VECTASHIELD mounting medium containing 4′, 6‐diamidino‐2‐phenylindole (DAPI) (Vector Laboratories Inc., Burlingame, CA) and stored at 4°C until image collection. Immunofluorescence images were obtained using a ZEISS LSM 510META equipped with C‐Apochromat 63x/1.2W lens (Carl Zeiss AG, Oberkochen, Germany).

### Determination of the levels of active GTP‐bound RhoA and total RhoA

The levels of active GTP‐bound RhoA and total RhoA were examined using the G‐LISA^®^ Active RhoA Activation Assay Biochem kit (Cytoskeleton Inc., Denver, CO) and the Total RhoA ELISA Biochem Kit (Cytoskeleton Inc.), respectively, according to the manufacturer's instruction. The human lung fibroblast cells were seeded at a density of 1 × 10^6^ cells on a 10 cm dish and cultured until approximately 90% confluence. Then, cells were serum‐starved for 24 h, followed by 2‐h stimulation with 10% FCS‐containing medium, the same culture condition for immunofluorescence and confocal microscopy. The cell lysates for RhoA assay were prepared as follows. Culture dishes were put on ice, washed with cold phosphate‐buffered saline (PBS), and lysed with an ice‐cold cell lysis buffer with protease inhibitor. The levels of RhoA activity and total RhoA were measured using a Benchmark Plus Microplate Reader (Bio‐RAD, Hercules, CA) detecting absorbance at 490 nm. The data are presented as both RhoA‐GTP and RhoA‐GTP/total RhoA ratio.

### Statistical analysis

Results are expressed as the means ± SD. Grouped data were evaluated by one‐way analysis of variance (ANOVA), and post hoc analyses were performed by Dunnett's multiple comparisons or multiple *t*‐test adjusted by Bonferroni's method. Continuous variables between two groups were assessed by Student's *t*‐test. Comparisons were considered statistically significant if *P* values were <0.05.

## Results

### Clinical characteristics of study population with BHDS

Lung fibroblasts were isolated and placed in primary cultures originated from 12 patients with BHDS, including seven men and five women. The average age at the isolation of lung fibroblast was 45.6 ± 9.4 years old (yo) (mean ± SD) (range, 34–62 yo). As a control, lung fibroblasts were also isolated from the normal parts of lung tissues from 10 patients with lung cancer (four men and six women) who had undergone a lobectomy for tumor removal. Their average age at the time of resection was 50.7 ± 14.8 yo (range, 33–78) and did not differ significantly from that of BHDS patients (*P* = 0.49). Six different germline *FLCN* mutations were identified and 11 of 12 patients had pulmonary manifestation alone among three major organs usually involved in BHDS (Table 1). We confirmed that lung fibroblasts used in this study were in a status of *FLCN* haploinsufficiency; (1) they carried wild‐type *FLCN* and the mutated *FLCN* with an identical abnormality that was originally found in peripheral blood leukocytes at the diagnosis of BHDS and (2) no loss of heterozygosity (LOH) was identified in the *FLCN* gene‐associated region (chromosome 17p12.2).

### Expression of folliculin, chemotaxis, and 3‐D gel contraction in BHDS lung fibroblasts

**Figure 1:**
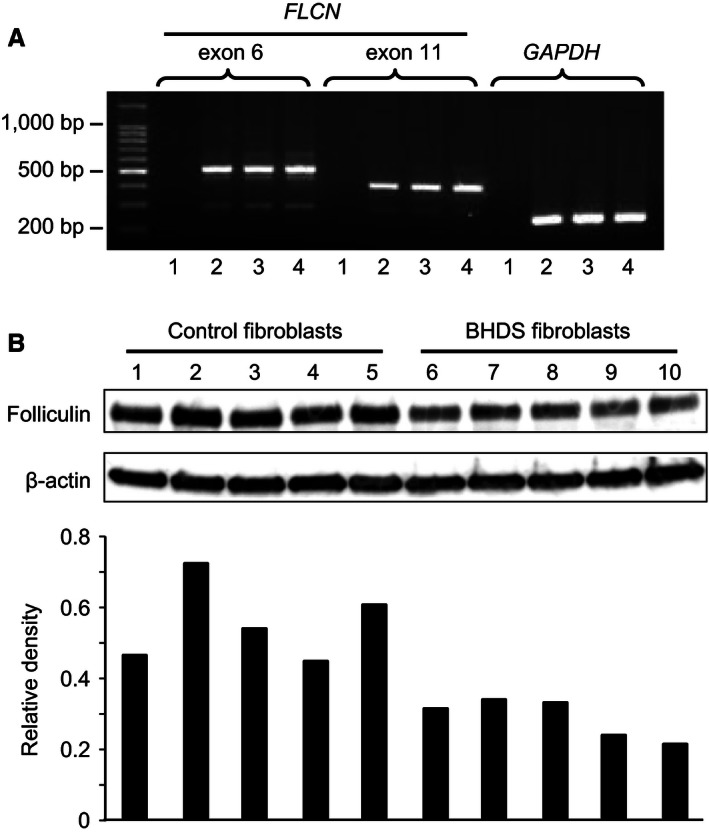
*FLCN* expression in lung fibroblasts. (A) *FLCN* expression was examined by RT‐PCR in human BHDS lung fibroblasts (lane 2, patient No. 4 in Table 1 as a representative of BHDS), human control lung fibroblasts (lane 3), and human fetal lung fibroblast (HFL‐1) cells (lane 4). Lane 1 was the negative control of RT‐PCR in which no cDNA was included in the reaction mixture. Two sets of primers were utilized for analysis: one set to amplify part of *FLCN* mRNA transcript contained mainly exon 6 and the one set for exon 11. Part of *GAPDH* mRNA transcript was amplified by RT‐PCR as a quality control for cDNA. (B) Folliculin levels were measured by western blot analysis in control lung fibroblasts (lanes 1–5) and BHDS lung fibroblasts (lane 6, patient no. 2 in Table 1; lane 7, patient no. 3; lane 8, patient no. 7; lane 9, patient no. 10; and lane 10, patient no. 11). The lower panel showed the relative intensity of folliculin as compared to that of *β*‐actin determined by densitometric quantification. Note that expression of folliculin was diminished in BHDS lung fibroblasts as compared to that of control lung fibroblasts (mean ± SD of the relative density: control vs. BDHS, 0.55 ± 0.1 vs. 0.28 ± 0.05, *P* < 0.001).

*FLCN* expression was confirmed by RT‐PCR in human BHDS lung fibroblasts (Fig. 1A). Folliculin levels were measured by western blot analysis in control lung fibroblasts and BHDS lung fibroblasts (Fig. 1B); protein expression of folliculin was markedly diminished in BHDS lung fibroblasts as compared to that of control lung fibroblasts (approximately, 51% of that of control fibroblasts). These results indicate that BHDS lung fibroblasts have *FLCN* haploinsufficiency and coincide with the results of genetic analysis.

**Figure 2:**
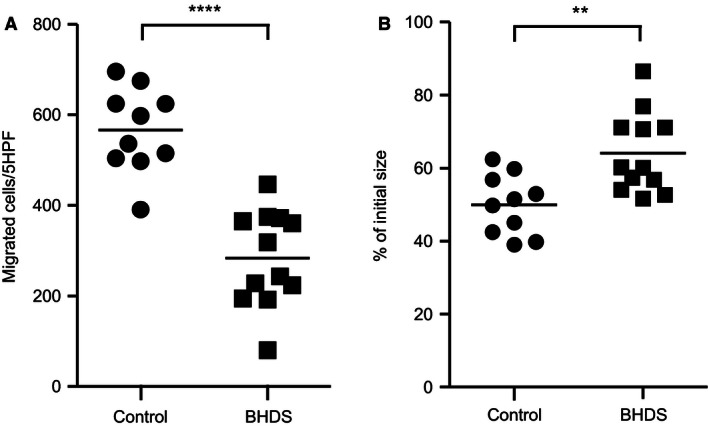
Chemotaxis and 3‐D collagen gel contraction by lung fibroblasts. Chemotactic activity toward fibronectin (20 μg/mL) (A) and 3‐D collagen gel contraction (B) by lung fibroblasts were assessed (control, *n* = 10 and BHDS,* n* = 12). The vertical axis in Figure 2A shows the number of migrated cells per five high‐power fields (5HPF), whereas the one in Figure 2B indicates the gel size expressed as a percentage of the original size 3 days after the initiation of assay. The horizontal line in each figure indicates the position of mean value.

Next, we evaluated the functions of lung fibroblasts considered to be important for the tissue repair response in control and BHDS. First, lung fibroblasts were grown in monolayer culture and assessed for chemotactic activity toward fibronectin (20 μg/mL) (control, *n* = 10 and BHDS, *n* = 12). We found that BHDS lung fibroblasts were less active in migration toward fibronectin as compared to control lung fibroblasts (Fig. 2A): on average, 566 ± 24 fibroblasts from controls migrated per five high‐power fields (5HPF) versus 284 ± 30 fibroblasts from BHDS migrated per 5HPF (*P* < 0.01). Second, we examined the extent of 3‐D collagen gel contraction: the gel size was expressed as a percentage of the original size at 3 days after the initiation of assay. Control lung fibroblasts embedded in 3‐D collagen gels contracted the gels to 50.0 ± 2.6% of the original gel size. In contrast, BHDS lung fibroblasts contracted the gels to 64.1 ± 3.2% of the original size (*P* < 0.01) (Fig. 2B).

### Expression of TGF‐*β*1 and extracellular matrix protein by BHDS lung fibroblasts

**Figure 3:**
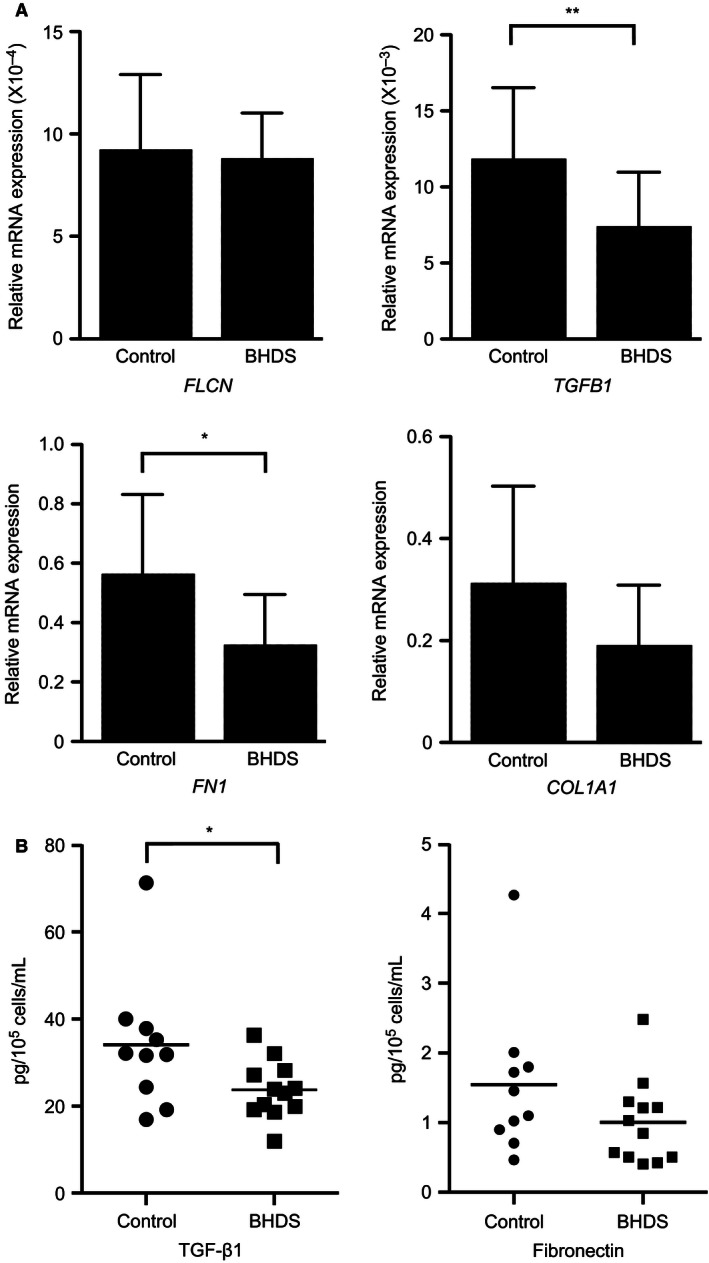
Expression of TGF‐*β*1 and fibronectin were diminished in BHDS fibroblasts. (A) qRT‐PCR of *TGFB1, FN1, COL1A1,* and *FLCN* expression in human control lung fibroblasts (*n* = 10) and BHDS lung fibroblasts (*n* = 12). Relative expression of each was calculated against the expression of the *GAPDH* gene. (B) Release of TGF‐*β*1 and fibronectin by cultured lung fibroblasts (control, *n* = 10 and BHDS,* n* = 12). Culture medium was harvested from 3‐D collagen gel contraction assay and then the concentrations of TGF‐*β*1 and fibronectin were evaluated by ELISA. The horizontal line in each figure indicates the position of mean value.

To evaluate the tissue‐repairing ability of lung fibroblasts, we examined mRNA expression of the several genes, *TGFB1* (TGF‐*β*1), *FN1* (fibronectin), and *COL1A1* (collagen 1), in control (*n* = 10) and BHDS lung fibroblasts (*n* = 12). The relative expression of each gene was calculated against the gene expression level of *GAPDH*. There was no significant difference in *FLCN* expression between control and BHDS fibroblasts (Fig. 3A). However, the expression of *TGFB1* and *FN1* were significantly diminished in BHDS lung fibroblasts (*P* < 0.01), although *COL1A1* (collagen 1) expression was only marginally diminished (*P* = 0.11) (Fig. 3A). We next measured the release of TGF‐*β*1 and fibronectin by cultured lung fibroblasts in 3‐D collagen gel. BHDS fibroblasts released a significantly smaller amount of TGF‐*β*1 than control fibroblasts (24.3 ± 1.3 pg/10^5^ cells/mL vs. 33.5 ± 3.2 pg/10^5^ cells/mL, *P* < 0.05). The quantity of fibronectin released from BHD fibroblasts tended to be lower than that from control fibroblasts (1.0 ± 0.1 pg/105 cells/mL vs. 1.46 ± 0.2 pg/105 cells/mL, *P* = 0.07) (Fig. 3B).

### Effect of knockdown of *FLCN* on the function of normal fetal lung fibroblasts (HFL‐1)

**Figure 4:**
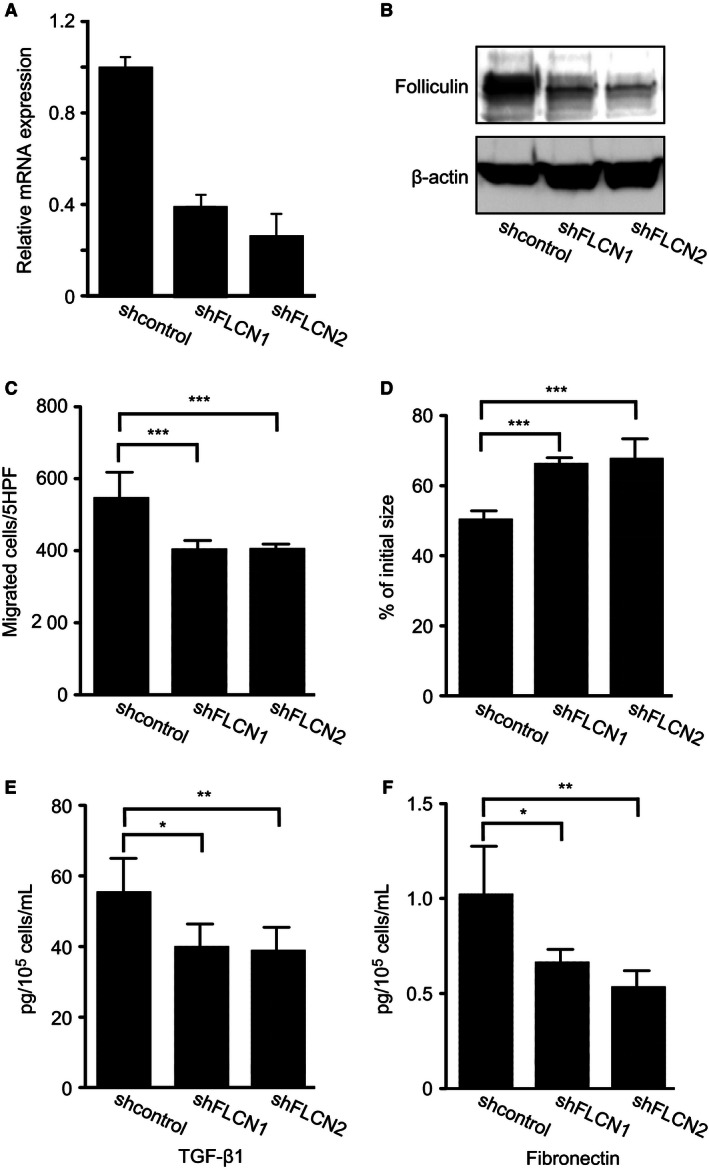
Chemotaxis, 3‐D collagen gel contraction, and the release of TGF‐*β*1 and fibronectin by HFL‐1 when *FLCN* expression was knocked down by shRNA. *FLCN* expression was knocked down in HFL‐1 cells by transfecting them a lentivirus that expresses shRNA against *FLCN* (shFLCN1 or shFLCN2). Irrelevant shRNA was transfected as a control (shcontrol). (A) *FLCN* mRNA level was measured by qRT‐PCR. Relative expression against the basal *FLCN* expression level in HFL‐1 cells is shown in a vertical axis. (B) Western blot analysis confirmed that folliculin expression in HFL‐1 was diminished by shFLCN1 and shFLCN2. Chemotactic activity toward fibronectin (20 μg/mL) decreased in HFL‐1 cells (C) and contraction of 3‐D collagen gels (D) were significantly suppressed in HFL‐1 cells when *FLCN* expression was knocked down. The vertical axis in Figure 4C and D resemble those of Figure 2A and B, respectively. The release of TGF‐*β*1 (E) and fibronectin (F) into the culture media harvested from 3‐D collagen gel cultures was significantly reduced. Grouped data were evaluated by ANOVA, and post hoc analyses were performed by Dunnett's multiple comparison.

Because less folliculin was expressed by BHDS lung fibroblasts than by those from controls, we sought to examine if knockdown of *FLCN* expression in normal fibroblasts results in the functional consequences like those of BHDS lung fibroblasts. *FLCN* expression was knocked down in HFL‐1 cells by infecting them with the lentivirus, which expresses shRNA against *FLCN* (shFLCN1 or shFLCN2). Reductions of both *FLCN* mRNA expression and protein production were confirmed by qRT‐PCR and western blot analysis, respectively (Fig. 4A and B). We next examined whether the fibroblast function was impaired by knockdown of *FLCN* in HFL‐1 cells and found that those knocked down cells exhibited reductions of not only chemotaxis but also 3‐D collagen gels contraction (Fig. 4C and D). In addition, the release of TGF‐*β*1 and fibronectin decreased significantly when *FLCN* expression in HFL‐l was knocked down (Fig. 4E and F, respectively).

### Effect of expression of naturally occurring mutated *FLCN* on the function of HFL‐1

**Figure 5:**
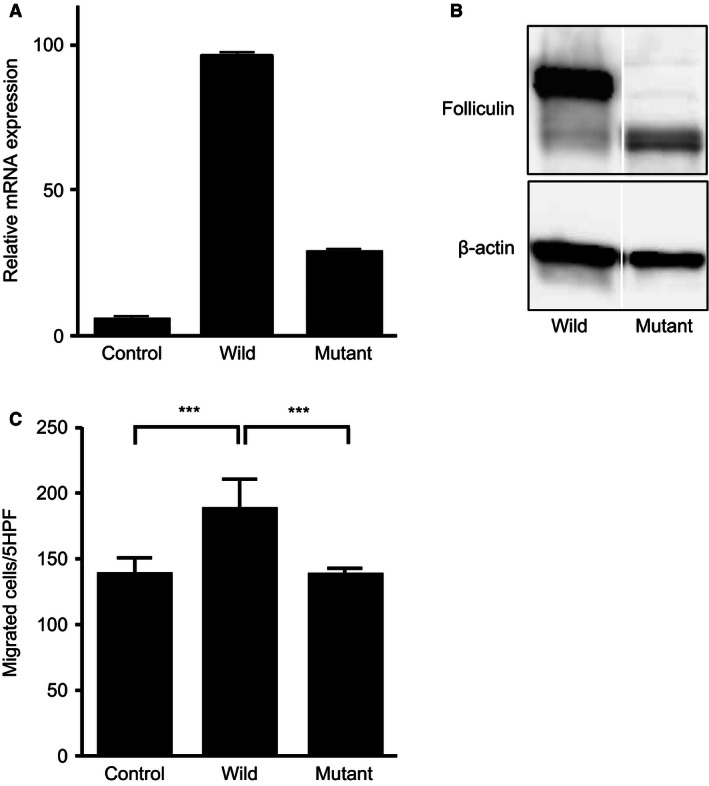
Transduction of FLAG‐tagged wild‐type or mutated *FLCN* into HFL‐1 cells by utilizing a lentivirus vector. (A) *FLCN* mRNA levels were measured by qRT‐PCR in HFL‐1 cells after transduction of a wild‐type (wild) or mutated *FLCN* (c.1285dupC) (mutant), or a vehicle lentivirus (control). The vertical axis indicates the relative *FLCN* expression in HFL‐1 cells. (B) Protein expression was examined by western blot analysis using a mouse monoclonal anti‐FLAG or anti‐*β*‐actin antibody, respectively. These samples were run on the same blot but nonessential lanes were removed. (C) Chemotactic activity toward fibronectin (20 μg/mL) showed that overexpression of wild‐type *FLCN* significantly increased chemotaxis, whereas no effect on chemotaxis followed the transduction of mutated *FLCN* (c.1285dupC) (C). The vertical axis depicted is similar to that of Figure 2A. Grouped data were evaluated by ANOVA, and post hoc analyses were performed by multiple t‐test adjusted by Bonferroni's method.

To evaluate the effect of *FLCN* mutation on fibroblast functions, FLAG‐tagged wild‐type or mutant *FLCN* cDNA was introduced into the HFL‐1 cells using a lentivirus vector. After infection with lentivirus carrying a wild‐type *FLCN*, mutated *FLCN* (c.1285dupC), or a vehicle lentivirus (control), *FLCN* mRNA levels were measured by qRT‐PCR in HFL‐1 cells. The expression of *FLCN* mRNA greatly increased in HFL‐1 cells when transfected by lentivirus carrying wild‐type (96.2 ± 0.06) and mutated *FLCN* (23.2 ± 0.04) as compared to those of vehicle lentivirus (Fig. 5A). Western blot analysis of transfected HFL‐1 cells then confirmed the marked production of FLAG‐tagged wild‐type folliculin and mutated protein, respectively (Fig. 5B). Chemotactic activity of HFL‐1 cells toward fibronectin significantly increased by overexpression of FLAG‐tagged wild‐type *FLCN*, although no effect on chemotaxis followed the induced expression of FLAG‐tagged mutated *FLCN* (c.1285dupC), suggesting that the mutant folliculin did not exert a dominant negative effect (Fig. 5C).

### Effect of TGF‐*β*1 on the function of *FLCN*‐knockdown HFL‐1 and BHDS lung fibroblasts

**Figure 6:**
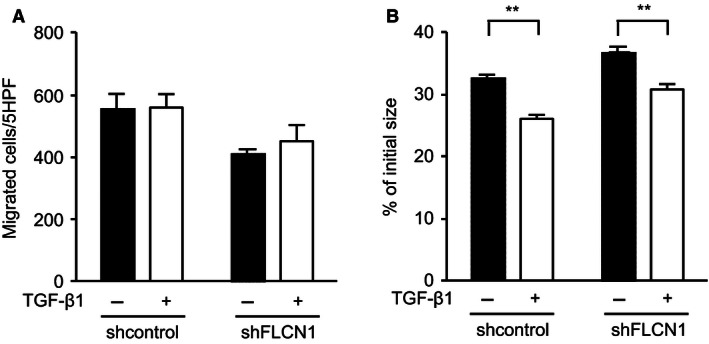
TGF‐*β*1 partially abrogates the effect of *FLCN* knockdown on chemotaxis and contraction of 3‐D collagen gels in HFL‐1 cells. HFL‐1 cells were transfected by a lentivirus that expressed shRNA against *FLCN* (shFLCN1) or control shRNA (control). *FLCN‐*knockdown reproducibly impaired both chemotaxis and gel contraction by HFL‐1 (*P* < 0.01). After transduction, HFL‐1 cells were maintained in either complete media without (−) or with TGF‐*β*1 (+) at 10 pmol/L and then subjected to assays for chemotaxis (A) and 3‐D collagen gel contraction (B) assays.

**Figure 7:**
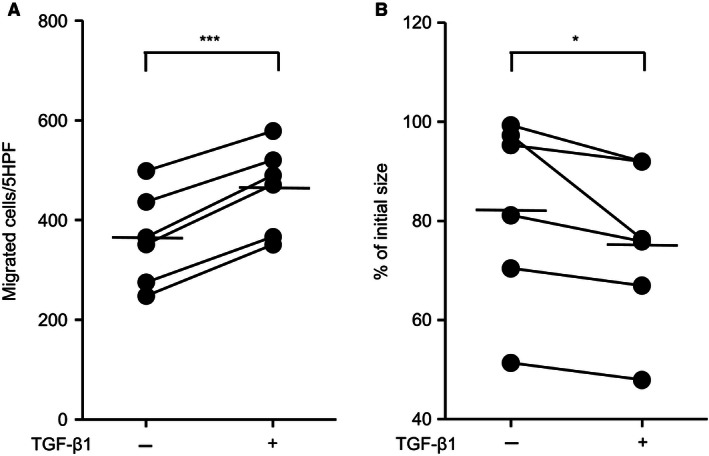
TGF‐*β*1 enhances chemotaxis and contraction of 3‐D collagen gels in BHDS lung fibroblasts. Chemotaxis (A) and contraction of 3‐D collagen gels (B) were evaluated in BHDS lung fibroblasts (*n* = 6) with (+) or without (−) TGF‐*β*1 at 10 pmol/L (see Methods S1); patients No. 6, 7, 8, 10, 11, and 12 in Table 1 were examined. The horizontal line in each figure indicates the position of mean value.

Presumably, the repair responses of fibroblasts declined, since the release of TGF‐*β*1 was decreased by knockdown of *FLCN*. We examined whether *FLCN*‐knockdown HFL‐1 cells could increase repair responses when exposed to exogenous TGF‐*β*1. However, no significant increase in chemotaxis was found in *FLCN*‐knockdown HFL‐1 as well as shcontrol‐transduced HFL‐1 when exposed to exogenous TGF‐*β*1 (Fig. 6A). In contrast, both control and *FLCN*‐knockdown HFL‐1 augmented 3‐D collagen gel contraction when exposed to exogenous TGF‐*β*1 (Fig. 6B). When we examined the effect of TGF‐*β*1 on BHDS lung fibroblasts, exogenously added TGF‐*β*1 effectively enhanced both chemotaxis and 3‐D collagen gel contraction (Fig. 7). We postulate that chemotaxis and gel contraction are impaired promptly in HFL‐1 after *FLCN*‐knockdown, but their chemotactic responsiveness to TGF‐*β*1 might need a longer period of folliculin‐deficient state like BHDS lung fibroblasts.

### Actin stress fibers and RhoA activity in BHDS lung fibroblasts

**Figure 8:**
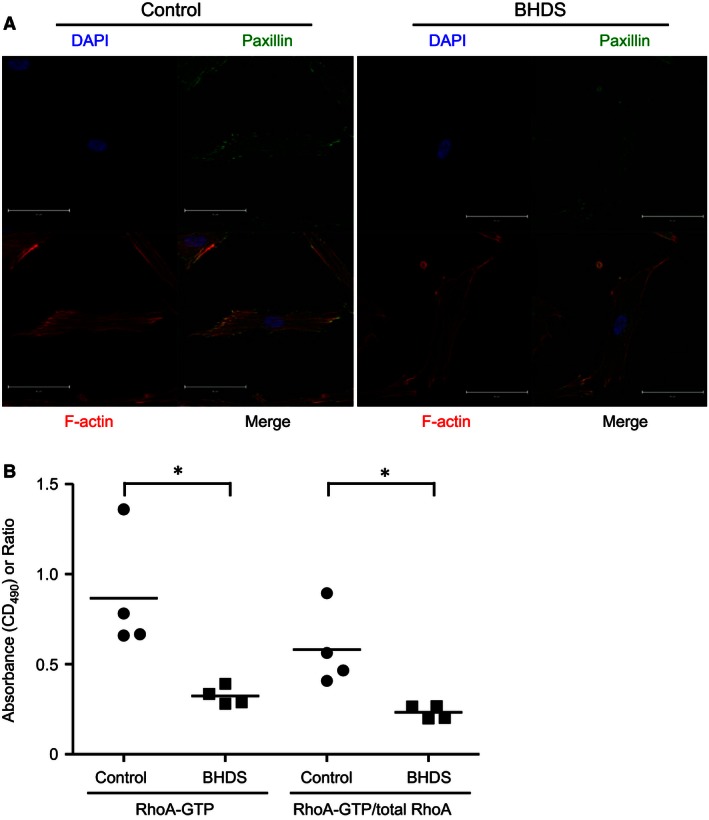
Organization of actin stress fiber, the association of actin with paxillin, and RhoA activity are decreased in BHDS lung fibroblasts. (A) Representative confocal images of phalloidin and paxillin staining in lung fibroblasts are shown; the results of BHDS lung fibroblasts isolated from patient 2 harboring c.769_777delTCC (exon 7) mutation are presented. The immunofluorescence staining was performed in both control (*n* = 4) and BHDS (*n* = 4: patients 2, 7, 10, and 11). Scale bars: 50 μm in each panel. (B) The levels of GTP‐bound RhoA (Rho‐GTP) and RhoGTP/total RhoA ratio are decreased in BHDS lung fibroblasts (*n* = 4: patients 2, 7, 10, and 11) as compared with those of control lung fibroblasts (*n* = 4) (*P < 0.05).

The many preceded studies showed that TGF‐*β*1‐mediated activation of RhoA activity, a key protein of Rho family of small GTPases, is important for the regulation of actin cytoskeleton, gel contraction, migration, and also myofibroblast differentiation (Chen et al. 2006; Milara et al. 2012; Ji et al. 2014). Accordingly, we next investigated the organization of actin stress fibers and RhoA activity in BHDS lung fibroblasts. Phalloidin staining showed that organization of actin stress fiber decreased in BHDS lung fibroblasts as compared with normal lung fibroblasts (Fig. 8A). Additionally, the expression of paxillin, a component of focal adhesion complex, and its association with actin stress fibers decreased in BHDS lung fibroblasts (Fig. 8A). When we examined the levels of GTP‐bound RhoA (Rho‐GTP) and RhoGTP/total RhoA ratio, both were diminished in BHDS lung fibroblasts as compared with those of control lung fibroblasts (Fig. 8B) (*P* < 0.05). These results suggest that long‐lasting haploinsufficiency state of the *FLCN* gene in patients with BHDS causes lung fibroblast dysfunction through impaired TGF‐*β*1/RhoA signaling pathway.

## Discussion

Here, BHDS lung fibroblasts harboring the state of *FLCN* haploinsufficiency, had notably diminished abilities to migrate, contract, and produce extracellular matrix proteins. As we demonstrated, these phenomena did not result from a dominant negative effect of mutated folliculin, but instead, was caused by the reduced amount of wild‐type folliculin protein itself. We found that knockdown of *FLCN* expression in HFL‐1 cells mimics the BHDS fibroblasts in terms of chemotaxis to fibronectin and ability of contraction in 3‐D collagen gel. When we transduced FLAG‐tagged wild‐type or mutant *FLCN* into HFL‐1 cells, the consequent overexpression of wild‐type *FLCN* significantly increased chemotaxis; however, the transduction of mutated *FLCN* (c.1285dupC) had no effect on chemotaxis. The clinical experience of patients with BHDS, whose germline *FLCN* mutation is expected to result in complete shut‐down of one *FLCN* allele without generating mutated aberrant proteins (Kunogi et al. 2010; Benhammou et al. 2011), supports this notion that the amount of folliculin protein rather than mutant protein is important.

Since lung fibroblasts are crucial for the maintenance of integrity of alveolar structure (Dunsmore and Rannels 1996), our results were expected to reveal impaired tissue repair responses in the lungs of BHDS patients whose peripheral alveoli underwent structural injury. When such injuries occur, chemotaxis, which is the ability of cells to migrate in response to a chemotactic gradient, is believed to be the means of recruiting new cells to damaged sites (Behr et al. 1993). Contraction of 3‐D collagen gels has been used for several decades as a model of the tissue contraction that characterizes the resolution of tissue granulation and the development of fibrosis (Montesano and Orci 1988; Kobayashi et al. 2006; Togo et al. 2008). In this process, mesenchymal cells attach to collagen fibers through integrin‐dependent mechanisms and generate mechanical tension. This mechanical tension results in tissue contraction, which typically reduces wound size. In addition, contraction has been associated with promoting apoptosis, and therefore, may play a role in the resolution phase of normal healing (Grinnell et al. 1999; Carlson and Longaker 2004; Kobayashi et al. 2005). Both chemotaxis and gel contraction bioassays to evaluate fibroblast function are utilized to model the cellular response to injury and subsequent tissue repair in vitro. For example, the lung fibroblasts from patients with COPD were shown to manifest reduced chemotaxis toward fibronectin and reduced contraction of 3‐D collagen gels, thus implicating this process as one mechanism contributing to the development of emphysema (Togo et al. 2008).

We found that expression of TGF‐*β*1 and fibronectin were diminished in the BHDS fibroblasts, suggesting that *FLCN* plays a role in the production of extracellular matrix proteins and cytokines relevant to tissue repair response. Furthermore, we demonstrated that the release of TGF‐*β*1 and fibronectin decreased in the *FLCN*‐knockdown cell line. Since TGF‐*β*1 induces fibroblasts to migrate and to synthesize extracellular matrix proteins, this cytokine has long been believed to be a key mediator in normal tissue repair and in the development of fibrosis (Sime et al. 1997; Gauldie et al. 2002; Leask and Abraham 2004). TGF‐*β*1 directly stimulates fibroblasts to produce increased amounts of extracellular matrix, including fibronectin and collagen (Yoshida et al. 1992; Lasky and Brody 2000). In addition, TGF‐*β*1 stimulates fibroblasts' chemotactic activity (Postlethwaite et al. 1987) and augments fibroblast‐mediated contraction of extracellular matrix (Montesano and Orci 1988), activities that may contribute to fibrosis (Kobayashi et al. 2006). An intimate association between folliculin and TGF‐*β* was previously reported. Hong et al. reported that several key genes involved in TGF‐*β* signaling, such as *TGFB2*,* INHBA*,* THBS1*, and *SMAD3*, were down‐regulated in *FLCN*‐null and mutant *FLCN* cells as well as in the BHDS‐associated renal tumors. Dysregulation of TGF‐*β* signaling by *FLCN* inactivation is likely to be an important step for tumorigenesis in BHDS (Hong et al. 2010), but we consider such abnormalities in TGF‐*β* signaling to be also implicated in pulmonary manifestations.

Several reports have proposed likely mechanisms for the development of pulmonary cysts in BHDS. Warren et al. demonstrated that *FLCN* mRNA was strongly expressed in stromal cells within the connective tissue and weakly in type I pneumocytes in the lung, proposing a possible role for functional abnormalities of folliculin‐expressing cells in cyst formation (Warren et al. 2004). Furuya et al. reported that BHD syndrome‐associated pulmonary cyst may be considered a hamartoma‐like cystic alveolar formation associated with deranged mTOR signaling (Furuya et al. 2012). Medvetz et al. discovered a physical interaction between folliculin and p0071, an armadillo repeat‐containing protein that localizes to the cytoplasm and to adherence junctions. They hypothesized that the defects in cell–cell adhesion in *FLCN*‐deficient cells underlie the pathogenesis of airspace enlargement in BHDS (Medvetz et al. 2012). In this context, our group has proposed a possible involvement of inherent tissue weakness due to *FLCN* haploinsufficiency, since the BHDS cysts develop in the periacinar region, an anatomically weak site in a primary lobule where alveoli attach to connective tissue septa (Kumasaka et al. 2014). Recently, Goncharova et al. reported that BHDS‐afflicted lungs exhibit increased alveolar epithelial cell apoptosis, alveolar enlargement, and an impairment of both epithelial barrier and overall lung function (Goncharova et al. 2014). Taken these clinical and experimental findings together into consideration, Kennedy et al. proposed the stretch hypothesis for pulmonary cyst pathogenesis: cysts in BHDS arise because of defects in cell–cell adhesion, leading to repeated respiration‐induced physical stretch‐induced stress and, over time, expansion of alveolar spaces particularly in the regions of lung with larger changes in alveolar volume and at weaker “anchor points” to connecting interlobular septa and pleura (Kennedy et al. 2016). We believe that the results of our present study add a new explanation for the mechanisms of cyst formation in the BHDS lung, diminished tissue repair responses driven by fibroblasts at those anatomically weak, vulnerable sites, that is, shear stress force in a primary lobule. Apoptosis of lung epithelial cells (Goncharova et al. 2014), weakened cell–cell interaction (Medvetz et al. 2012), and impaired fibroblast function appear to cooperate for the BHDS cysts to develop. Our histopathological analyses clearly showed no inflammation was present in intrapulmonary small cysts; instead, the role of inflammation, if implicated in BHDS cyst formation, resides in growing its size (Kumasaka et al. 2014). Still in need of clarification is what triggers the cyst formation that switches on these cooperative mechanisms.

## Conflict of Interest

The authors have reported that no potential conflicts of interest exist with any companies/organizations whose products or services may be discussed in the article.
